# Left ventricle segmentation in transesophageal echocardiography images using a deep neural network

**DOI:** 10.1371/journal.pone.0280485

**Published:** 2023-01-20

**Authors:** Seungyoung Kang, Sun Ju Kim, Hong Gi Ahn, Kyoung-Chul Cha, Sejung Yang

**Affiliations:** 1 Department of Biomedical Engineering, Yonsei University, Seoul, Korea; 2 Department of Emergency Medicine, Yonsei University Wonju College of Medicine, Wonju-si, Korea; Shandong Normal University, CHINA

## Abstract

**Purpose:**

There has been little progress in research on the best anatomical position for effective chest compressions and cardiac function during cardiopulmonary resuscitation (CPR). This study aimed to divide the left ventricle (LV) into segments to determine the best position for effective chest compressions using the LV systolic function seen during CPR.

**Methods:**

We used transesophageal echocardiography images acquired during CPR. A deep neural network with an attention mechanism and a residual feature aggregation module were applied to the images to segment the LV. The results were compared between the proposed model and U-Net.

**Results:**

The results of the proposed model showed higher performance in most metrics when compared to U-Net: dice coefficient (0.899±0.017 vs. 0.792±0.027, p<0.05); intersection of union (0.822±0.026 vs. 0.668±0.034, p<0.05); recall (0.904±0.023 vs. 0.757±0.037, p<0.05); precision (0.901±0.021 vs. 0.859±0.034, p>0.05). There was a significant difference between the proposed model and U-Net.

**Conclusion:**

Compared to U-Net, the proposed model showed better performance for all metrics. This model would allow us to evaluate the systolic function of the heart during CPR in greater detail by segmenting the LV more accurately.

## Introduction

Chest compression is a major component that facilitates systemic circulation during cardiopulmonary resuscitation (CPR). Optimal chest compression can promote good resuscitation outcomes in patients with cardiac arrest. Although recent CPR guidelines recommend using a real-time feedback device to maintain high-quality CPR during resuscitation, it has rarely been associated with favorable resuscitation outcomes [1–3]. One of the reasons might be the unmonitored location of the chest compression. Previous studies based on chest computed tomography also observed that the currently recommended location of chest compression was too high to effectively compress the left ventricle (LV) [4, 5].

Transesophageal echocardiography (TEE) has been proposed as a good modality for identifying correctable causes of cardiac arrest and monitoring the quality and location of CPR [6–8]. It can also identify the heart structures compressed by external chest compression without interruption of the chest compression during resuscitation [9]. Thus, we can evaluate the exact location of chest compression and systolic function generated by external chest compression from TEE images acquired from cardiac arrest patients. This might verify the optimal chest compression methods for facilitating LV systolic function during CPR [10–12].

**Fig 1:**
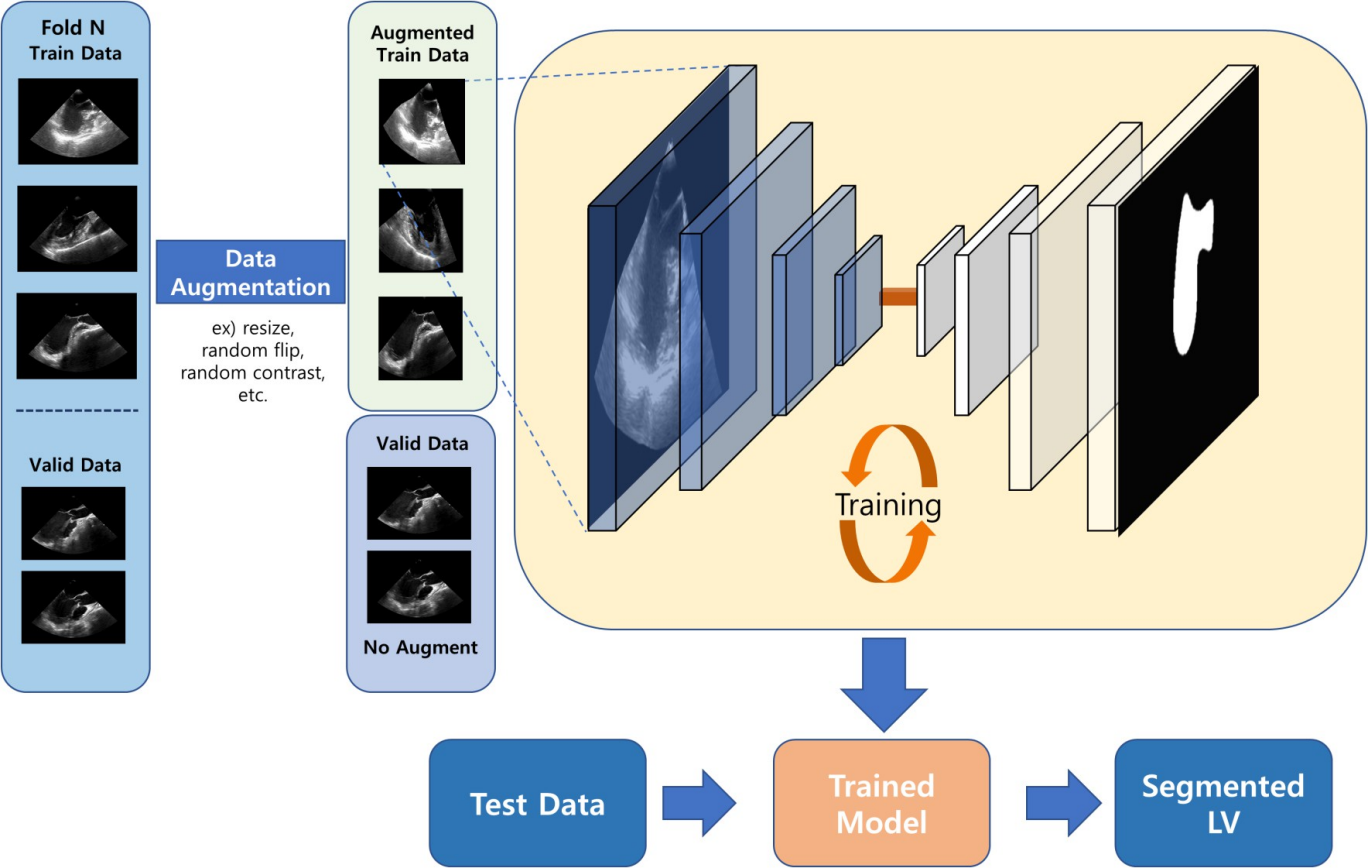
Workflow of LV segmentation in TEE images. The dataset was split into train, validation and test dataset. The training dataset was increased by the augmentation method, because of the limited data. LV, left ventricle; TEE, transesophageal echocardiography.

Segmenting the LV is necessary to determine the location of chest compression and obtain indicators for quantitative evaluation of heart function, such as end-diastolic volume, end-systolic volume, area, and ejection fraction. Many attempts have been made to segment the LV. Based on the contour tracking methodology, Noble et al., [13] adopted a Kalman-filter-based epicardial and endocardial boundary tracking system. Bosch et al. [14] improved active appearance models for boundary detection to the active appearance motion model for fully automated powerful and sequential detection of the LV. Most cardiac images, such as ultrasound images and magnetic resonance imaging (MRI), have ambiguous boundaries and severe noise; therefore, the analysis of these images take time, and the results may vary from person to person. Artificial neural networks have been proposed as they provide high analysis accuracy and make it possible to generalize medical images [15, 16]. Smistad et al. [17] suggested deep convolutional neural networks for LV segmentation model using U-Net [18], which consists of an encoder-decoder that shows a robust segmentation model in biomedical images. However, U-Net does not consider the contribution of all semantic features in the decoding procedure. Therefore, Moradi et al. [19] developed a modified U-Net called multi-feature pyramid U-Net, in which the features are complemented by linking feature maps at all levels of the U-Net decoder path. However, the existing methods have a limitation in that they are unable to recognize the ambiguous boundary between the shadow and LV. Moreover, TEE images acquired during CPR are noisier than normal echocardiograms due to chest compressions. We developed networks based on U-Net by applying the residual feature aggregation method and various attention techniques. Our model shows not only a strong feature extraction technique using a squeeze and excitation block and a residual block, but also focuses on the more important features. The workflow is shown in Fig 1. The following section describes the data organization, data augmentation techniques for deep learning, and the structure of our models.

## Materials and methods

### Dataset

The TEE images were obtained from dataset of Intra-Cardiac Arrest Research Using Sonography (ICARUS) registered at Wonju Severance Christian Hospital, Wonju, Republic of Korea. When patients with cardiac arrest arrived at the emergency department, CPR team performed advanced cardiac life support (ACLS) according to the CPR guideline. 20 Intra-arrest TEE was attempted if an emergency physician who was capable of performing TEE was available. An emergency physician, who was not participated in resuscitation performed TEE if return of spontaneous circulation was not obtained after approximately 10 min of ACLS. A multiplane TEE machine (EPIQ7; Philips Medical Systems, Andover, MA) was used to conduct TEE. Once TEE transducer (X8-2t 2–8 MHz transducer, Philips Medical System) was introduced into the esophagus, images were obtained according with the intra-arrest protocol including 4-chamber view, long axis view, 2-chamber view, aortic valve short axis view, ascending aorta short axis view, ascending aorta long axis view at the mid-esophageal level and descending aorta long axis view and aortic arch long axis view at the upper esophageal level. After quick observation of the heart and great vessels, the transducer was positioned at the mid-esophageal level to monitor cardiac movement and assist resuscitation measures. Since the TEE images were obtained during resuscitation, the heart was swung due to chest compression, which was different from normal beating heart. A part of the heart could not be evaluated during compression systole or diastole in 4-chamber view or 2-chamber view. Therefore, mid-esophageal long axis views were selected to precede image segmentation. The study design was approved by the Institutional Review Board of Wonju Severance Christian Hospital, Yonsei University (Approval number: CR317048). The requirement for obtaining informed consent was waived by the Institutional Review Board.

Nine patients’ data was collected; the patients were categorized as follows: six patients in a training dataset, two in a validation dataset, and one in a test dataset. To proceed with image segmentation using deep learning, there must be a mask, which is the result of image segmentation. Since masks for deep learning are usually made by humans, manufacturing masks take a lot of time. Moreover, making accurate masks for LV in TEE images in contact with severe noise is difficult without a clinician’s opinion. For this reason, it is difficult to create masks for all frames; therefore, we organized the data into frames corresponding to end-systole and end-diastole for every patient. Two experienced experts manually contoured to make ground truth for deep learning. When configuring data for deep learning, five-fold cross-validation was performed to use all datasets for evaluation and to prevent overfitting to a specific dataset. The distribution of the training data and validation data is shown in S1 and S2 Tables.

### Data augmentation

Data augmentation is a technique used to increase the amount of data required. There are many methods to perform data augmentation, but the essence is to artificially change the image. the number of training data per fold was small for deep learning. Since there was a high possibility of overfitting, we applied augmentation methods to our data. Excessive deformation by data augmentation can fade the meaning of the data; therefore, methods suitable for ultrasound images were applied. The applied data augmentation methods include random contrast control, random gamma control, horizontal flip, grid distortion, three-dimensional rotation, etc.

### Deep neural network architecture

**Fig 2:**
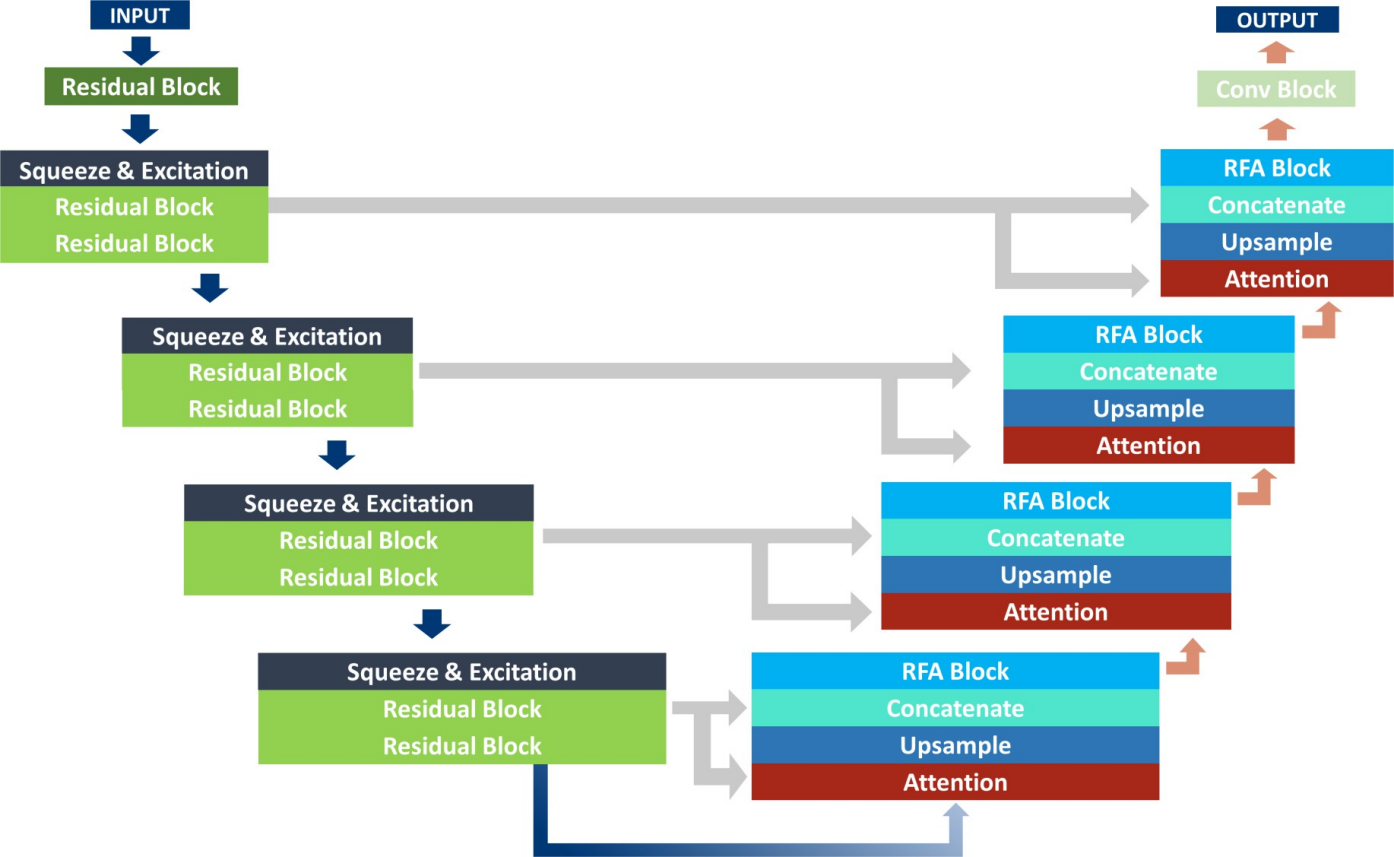
Proposed model architecture. The gray arrow indicates the skip connection that connects the encoder and decoder. The blue arrow indicates the encoding path and the red arrow indicates the decoding path. LV, left ventricle; TEE, transesophageal echocardiography.

We designed a deep neural network suitable for our data by appropriately placing residual blocks, residual feature aggregation modules, squeeze and excitation blocks, and attention blocks [20–23]. The proposed model is an encoder-decoder structure based on U-Net. The encoder step consists of four stages, each of which is composed of a squeeze and excitation block and two residual blocks. Residual blocks placed throughout the model prevent the vanishing gradient problem that occurs as the model goes deeper with residual mapping and the extraction of strong local features. The second residual block was used to adjust the stride to reduce the size of the feature map by half, which is called down-sampling. This not only reduces the number of parameters and computational resources, but also extracts spatial features. The extracted feature maps propagate to the next step while recalibrating the features as they pass the squeeze and excitation blocks. The attention block of the decoding stage gathers the features of the encoder and decoder steps to focus on the important features of the feature maps. Up-sampling was performed using a trainable convolution transpose filter, and spatial information was reflected as much as possible by collecting the features of the residual block using the residual feature aggregation module. In general, the process of down-sampling and up-sampling through the encoder and decoder can cause loss of information in the data. However, the skip connection that transfers the important information of the encoder step directly to the decoder minimizes the loss of information. Finally, the segmentation map of the TEE image was generated by propagating through the last convolution layer. The model that proposed in this study is depicted in Fig 2.

### Residual feature aggregation module

The residual feature aggregation (RFA) module consists of three residual blocks and three enhanced spatial attention (ESA) blocks. These residual blocks extract powerful features while retaining the original feature map using residual mapping. As shown in S1 Fig, the RFA module aggregates the local residual features of each residual block and reuses features to minimize the loss of information. The feature map passed through the RFA module is propagated to the last residual block and concatenated before the convolution layer. In addition, it maintains information about the original information by adding itself regardless of the residual mapping in each residual block. The ESA block behind the residual block utilizes a wide receptive field to focus on more important spatial features, and the features are multiplied by the feature map. This allows the model to learn more particular features, further enhancing model performance. The detailed structures of the residual block and the ESA block are shown in S2 Fig.

### Squeeze and excitation block

The squeeze and excitation block is one of the attention methods designed to enhance the representational power of convolutional features. This block is divided into two stages: squeezing and excitation. In the squeeze step, global spatial information is compressed into one channel through an average operation to extract important information for each channel. Then, in the excitation step, the compressed information from the previous step is recalibrated to highlight the important information with the channel-wise operation. The proposed model uses this block to focus on more important information when extracting features from the encoder step.

### Attention block

The attention method is primarily used to process natural language. Recently, this method has also been applied to image processing, such as image segmentation. The mechanism of the attention method in this model involves a second step. The first step compresses the important features of both the encoder and decoder. The second step is to resample the compressed information and multiply it to the original data to make the data more meaningful.

### Experiment detail

The proposed model was developed based on a deep learning framework called Pytorch. The original data size was resized from 600 × 800 to 256 × 256 to proceed with the learning. The batch size was set to 8, the learning rate was set to 0.00005, and the Adam optimizer was used to optimize the model. We applied a loss function, which is a mixture of binary cross-entropy loss, focal loss, and dice coefficient loss, unlike the original U-Net.

### Statistical analysis

In this study, the experiment was conducted with a different distribution of data through five-fold cross-validation. The metrics used for performance evaluation are the dice coefficient [24], intersection of union (IoU), recall, and precision. The dice coefficient and IoU are metrics that compare ground truth and predict results to indicate similarity; therefore, they are commonly used in image segmentation [25–27]. These metrics were statistically analyzed using 95% confidence intervals (CIs). Since the resulting metrics do not satisfy normality and homoscedasticity (S3 and S4 Figs), we implemented a Mann-Whitney U test between the proposed model and U-Net, under a significance level of 0.05. The details of metrics are described in S3 Table.

## Results

The data used in this study were TEE images obtained during CPR in nine patients. The acquired data were split into training, validation, and test datasets. As shown in Table 1, all metrics of the proposed model showed higher scores than U-Net. Groups were divided into the proposed model and U-Net, and a Mann-Whitney U test was performed for each metric. In the dice coefficient, the proposed model (Dice score: 0.899, 95% CI 0.875–0.909) and U-Net (Dice score: 0.792, 95% CI 0.770–0.823) showed a p-value less than 0.05, indicating significant differences between the groups. The two groups showed significant differences in terms of IoU and recall, with a p-values less than 0.05. The proposed model (Precision: 0.901, 95% CI 0.874–0.915) and U-Net (Precision: 0.859, 95% CI 0.829–0.896) showed no differences in precision, with a p-value of 0.571 (Table 2).

**Table 1:** Results of five-fold cross-validation for U-Net and the proposed model in the test dataset.

	U-Net				Proposed model			
	Dice coefficient	IoU	Recall	Precision	Dice coefficient	IoU	Recall	Precision
Fold#1	0.718	0.576	0.797	0.658	0.837	0.730	0.860	0.833
Fold#2	0.852	0.747	0.776	0.959	0.936	0.881	0.914	0.961
Fold#3	0.698	0.541	0.589	0.878	0.930	0.870	0.914	0.949
Fold#4	0.884	0.793	0.890	0.881	0.936	0.881	0.963	0.913
Fold#5	0.807	0.683	0.733	0.921	0.854	0.749	0.868	0.850
Mean	0.792	0.668	0.757	0.859	0.899	0.822	0.904	0.901

IoU, intersection of union

**Table 2:** Mann-Whitney U test results between U-Net and the proposed model.

	U-Net		Proposed model		p-value
	Mean	95% CI	Mean	95% CI	
Dice coefficient	0.792	0.770–0.823	0.899	0.875–0.909	< 0.05
IoU	0.668	0.640–0.708	0.822	0.786–0.838	< 0.05
Recall	0.757	0.726–0.799	0.904	0.875–0.921	< 0.05
Precision	0.859	0.829–0.896	0.901	0.874–0.915	0.571

CI, confidence interval; IoU, intersection of union

**Fig 3:**
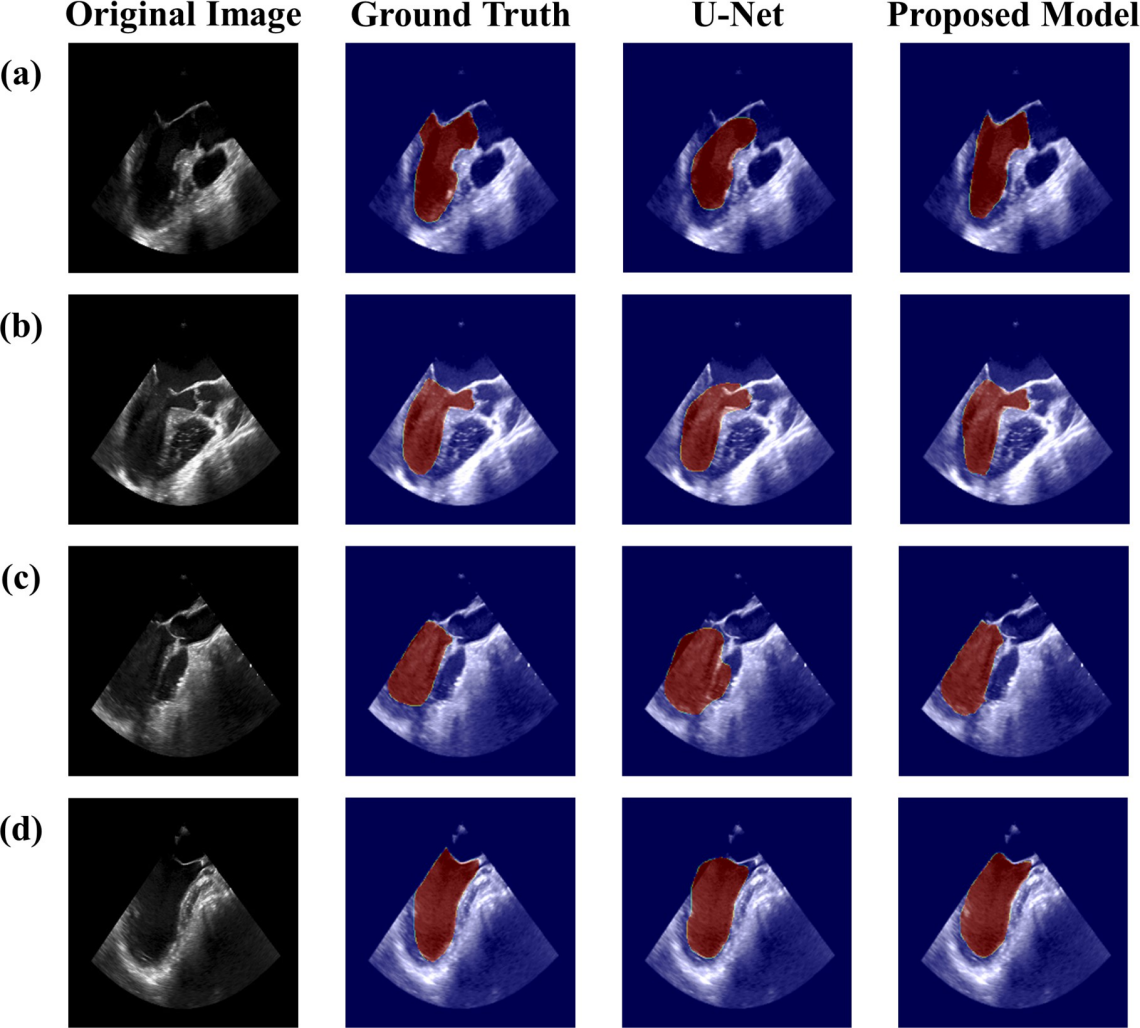
Comparison of the LV segmentation results between U-Net and the proposed model. LV, left ventricle.

Fig 3 shows a comparison of the segmentation map between the proposed model and U-Net. For U-Net, the segmentation map was distorted compared to the ground truth since the shadow of the TEE image was predicted as the boundary of the LV. In case of the proposed model, the boundary of the LV was well predicted, and the result was similar to the ground truth.

**Fig 4:**
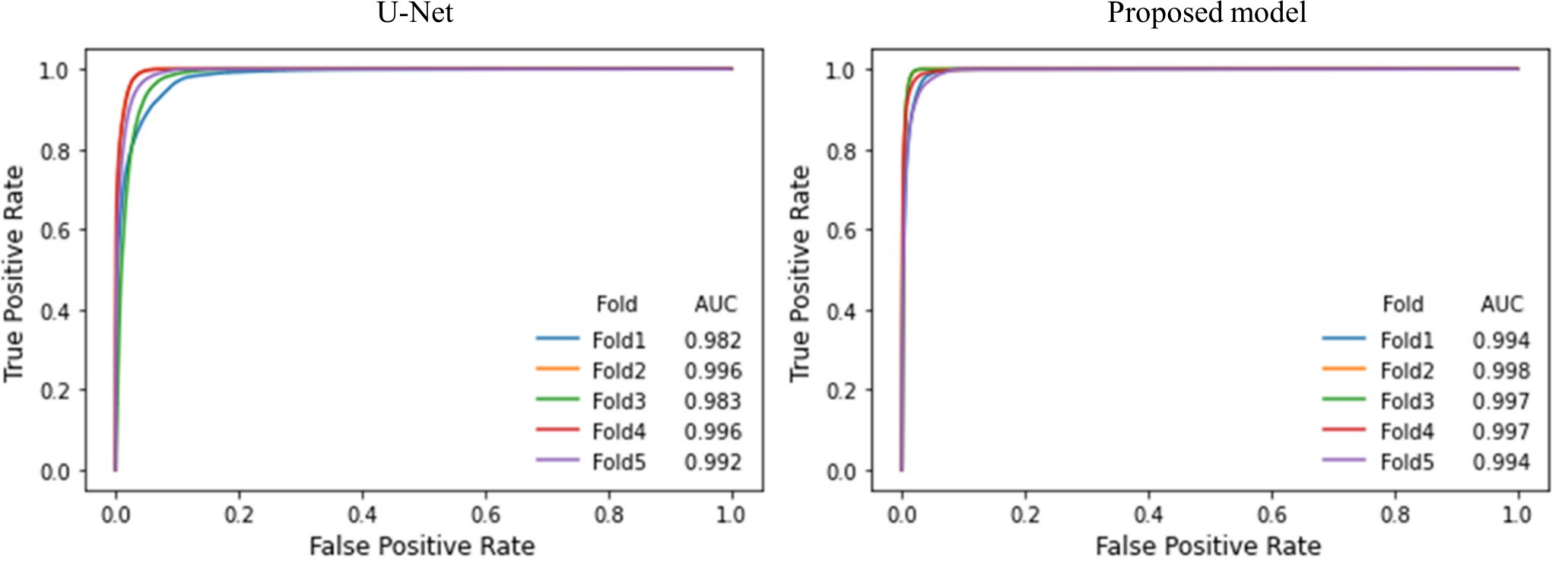
ROC curve analysis. The left and right sections of the image are the ROC curves of U-Net and the proposed model, respectively. ROC, receiver operating characteristic.

In Fig 4, U-Net predicted the LV with an area under the curve of 0.982, 0.996, 0.983, 0.996, and 0.992 in each fold. The proposed model predicted the LV with an area under the curve of 0.994, 0.998, 0.997, 0.997, and 0.994 in each fold. The proposed model showed better performance for all folds.

## Discussion

Segmenting the LV is necessary to determine the location of chest compression and acquire indicators for the quantitative evaluation of heart function. To date, many attempts have been made to segment the LV. These studies used medical images such as MRI, transthoracic echocardiogram, and TEE. However, the medical images used in this study were TEE images obtained during CPR. There was severe noise in the images, as they were obtained during chest compressions and there was also a frame in which the LV was pushed out of the scope. These images take time for clinicians to analyze, and the results may vary from person to person. To solve these problems, we designed a deep neural network using various deep learning techniques and applied it to this task.

The proposed model in the five-fold experiment showed better performance than U-Net in terms of the mean value of the dice coefficient, IoU, recall, and precision. Both the models were analyzed with a 95% CI and the proposed model had a narrower CI than U-Net. This implies that when repeated experiments were conducted, the results were distributed close to the mean, indicating that the model could reliably predict the LV. However, there was no difference between the two groups in terms of precision. U-Net is designed to judge the predicted value as LV, if it is 0.5 or more. The prediction results are distributed around 0.5; hence, the precision seems to be high as there are many true positive values. When conducting statistical analysis in other metrics, the dice coefficient, IoU, and recall showed significant differences between the two groups. The dice coefficient and IoU are the most popular indicators of image segmentation and are important performance evaluation indicators. As these two indicators are high, it means that the image segmentation result is similar to the ground truth. Therefore, it can be said that the proposed model showed better results in segmentation than U-Net. TEE is relatively non-invasive and can show high-resolution images without disturbing the CPR and the procedures performed during resuscitation. Hence, it has been an attractive modality for identifying correctable causes of cardiac arrest and monitoring the quality of CPR. These results can be applied to further studies to verify the optimal location of chest compression and augment cardiac output during CPR. In conclusion, we developed a new deep neural network for segmenting the LV. To segment the LV accurately, various attention methods and RFA modules were applied. With the abovementioned methods, we were able to achieve better segmentation results than U-Net; however, in some cases, we did not achieve robust results owing to noise and image quality. If we consider the preprocessing of noise or processing noise in the model, better segmentation results can be achieved in further studies.

## Supporting information

S1 Fig The residual feature aggregation block architecture. The residual features that pass the enhanced spatial attention block are merged into one place.
(TIF)

S2 Fig (a) Block diagram of the residual block. (b) Enhanced spatial attention block. These blocks are contained in the residual feature aggregation blocks.
(TIF)

S3 Fig The results of statistical tests for data normality (Shapiro-Wilks test). (TIF)

S4 Fig The results of statistical tests for data homoscedasticity (Bartlett’s test). (TIF)

S1 Table The distribution of training, validation, and test datasets. (DOCX)

S2 Table Data distribution of five-fold cross-validation for deep learning. (DOCX)

S3 Table Formula of the metrics. (DOCX)
