# Supplementary figures and images for: Left ventricle segmentation in transesophageal echocardiography images using a deep neural network
Source: PLoS One. 2023 Jan 20;18(1):e0280485. doi: 10.1371/journal.pone.0280485 (PMC9858054; doi:10.1371/journal.pone.0280485)

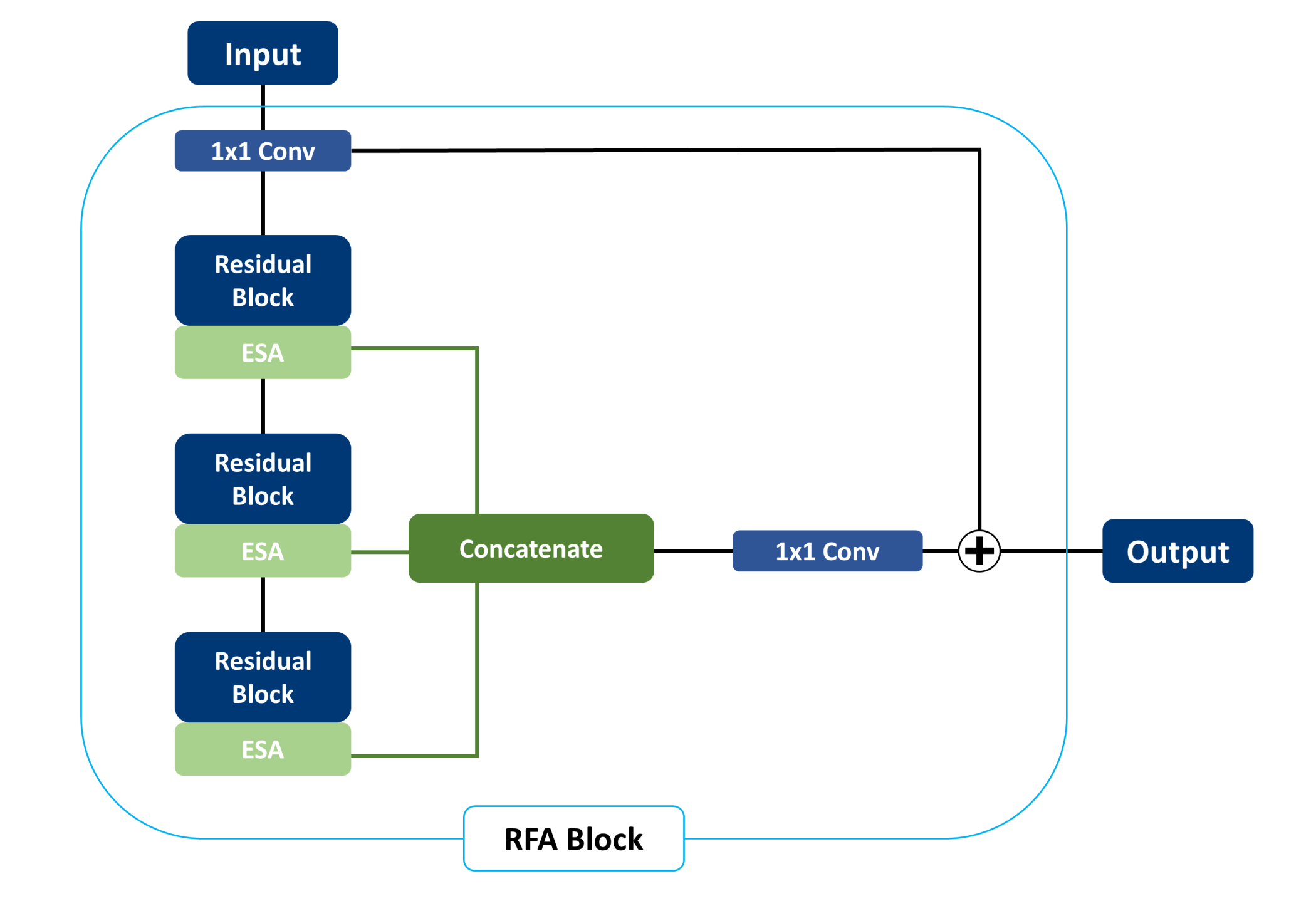

Supplement: S1 Fig — The residual features that pass the enhanced spatial attention block are merged into one place. (TIF) [file pone.0280485.s001.tif]

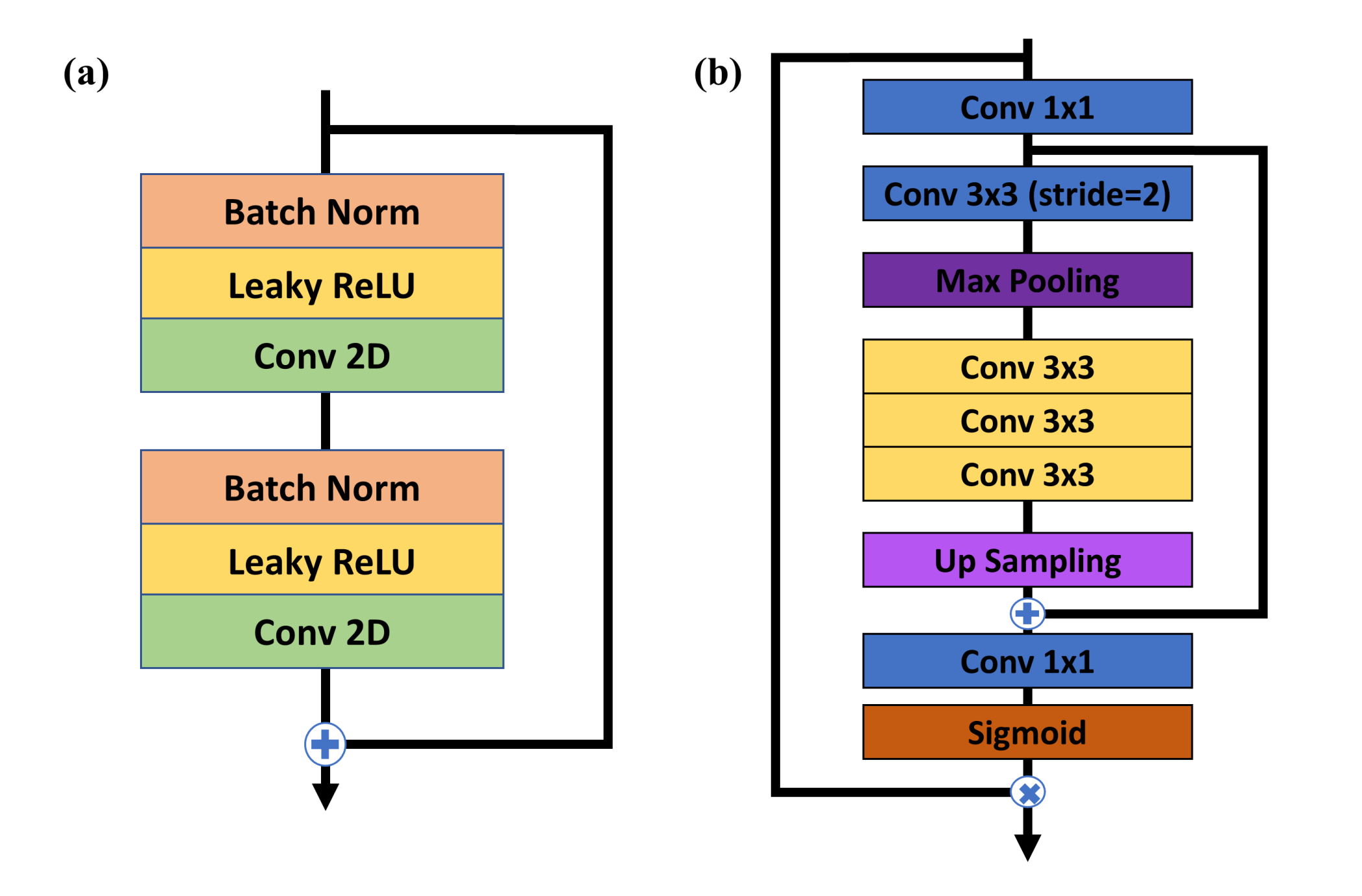

Supplement: S2 Fig — (a) Block diagram of the residual block. (b) Enhanced spatial attention block. These blocks are contained in the residual feature aggregation blocks. (TIF) [file pone.0280485.s002.tif]

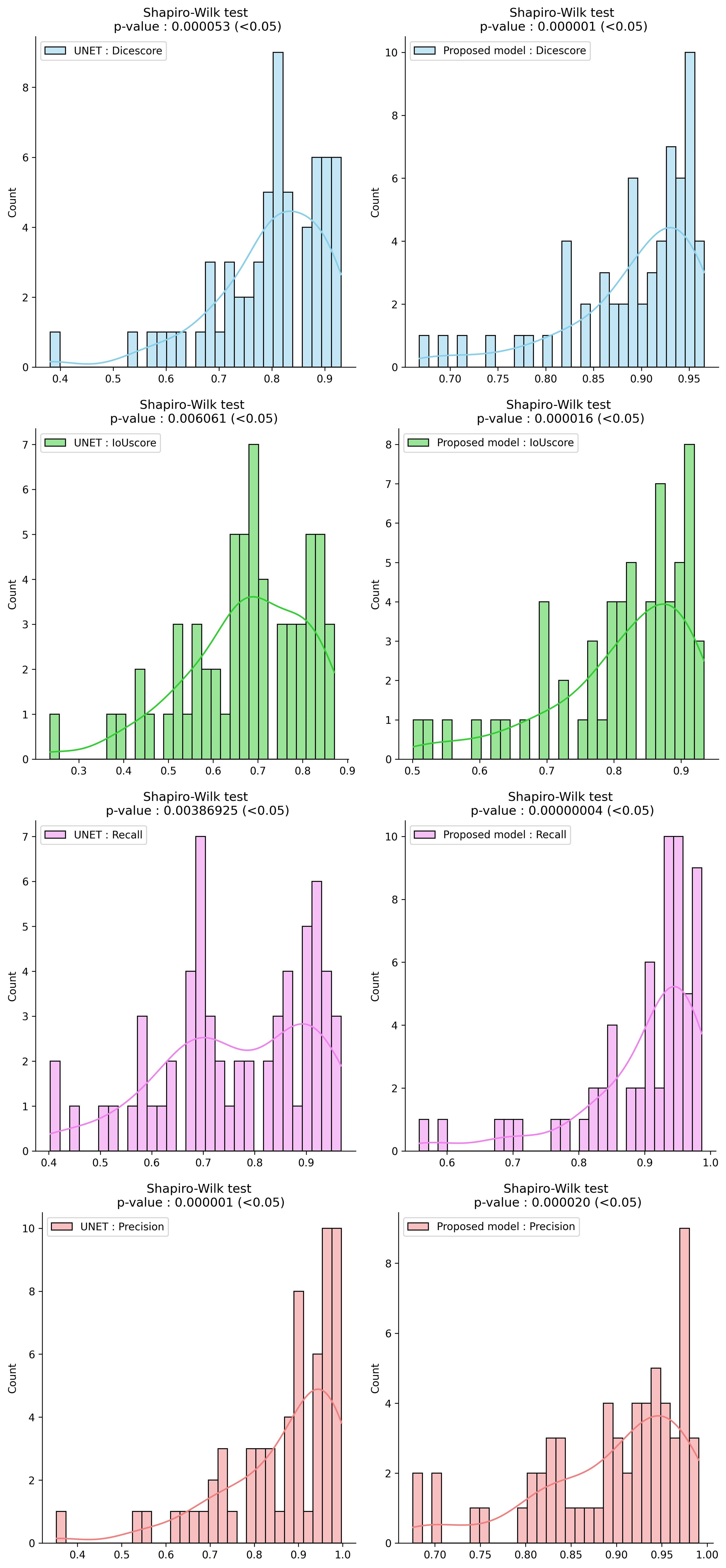

Supplement: S3 Fig — (TIF) [file pone.0280485.s003.tif]

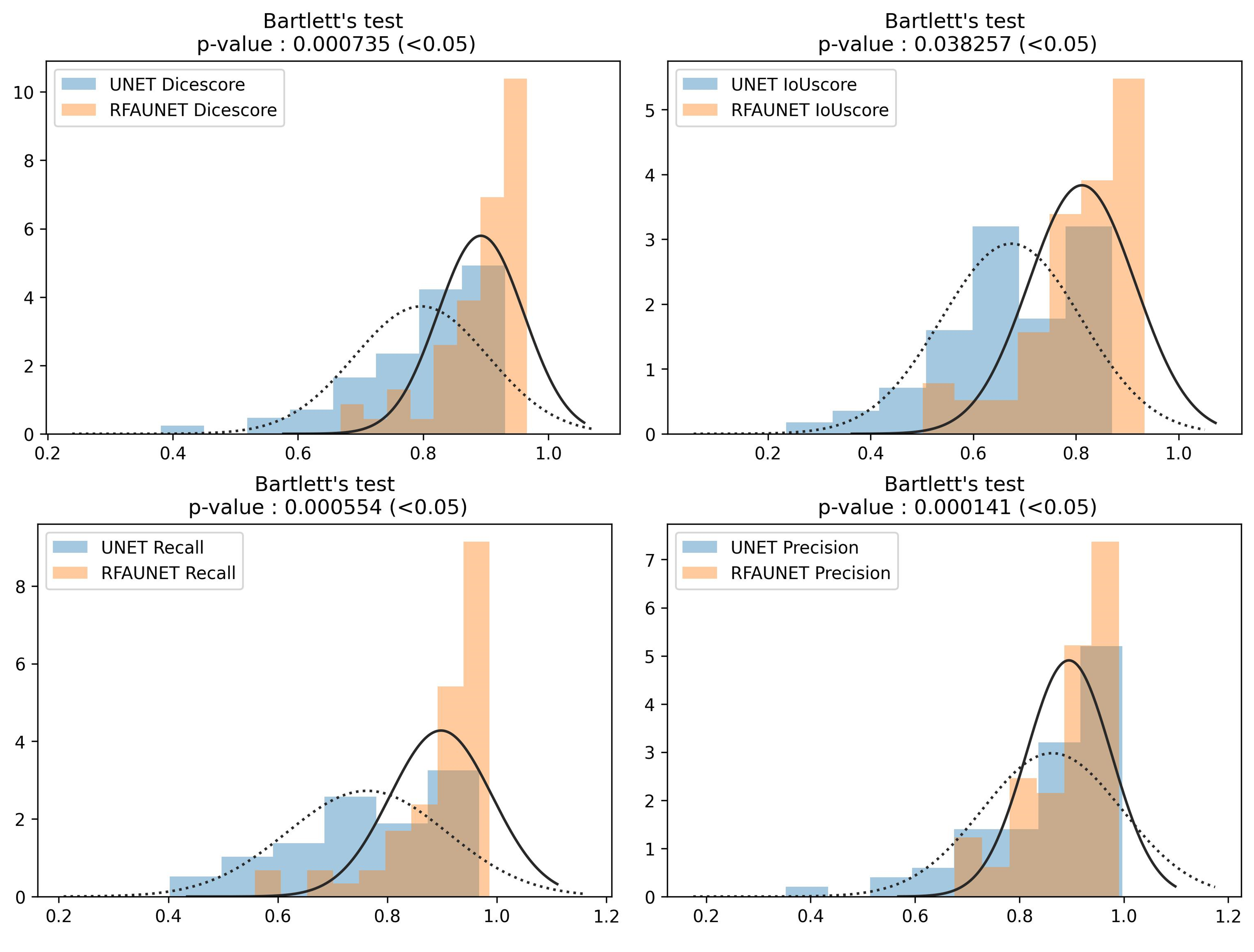

Supplement: S4 Fig — (TIF) [file pone.0280485.s004.tif]
