# Supplementary material for: Left ventricle segmentation in transesophageal echocardiography images using a deep neural network
Source: PLoS One. 2023 Jan 20;18(1):e0280485. doi: 10.1371/journal.pone.0280485 (PMC9858054; doi:10.1371/journal.pone.0280485)
Supplement: S1 Table — (DOCX) [file pone.0280485.s005.docx]

**S1 Table.** The distribution of training, validation, and test datasets.

|  | Trian data | Validation data | Test data |
| --- | --- | --- | --- |
| Fold#1 | 85 | 22 | 13 |
| Fold#2 | 88 | 18 | 14 |
| Fold#3 | 89 | 23 | 8 |
| Fold#4 | 80 | 30 | 10 |
| Fold#5 | 77 | 26 | 17 |
