# Supplementary material for: Left ventricle segmentation in transesophageal echocardiography images using a deep neural network
Source: PLoS One. 2023 Jan 20;18(1):e0280485. doi: 10.1371/journal.pone.0280485 (PMC9858054; doi:10.1371/journal.pone.0280485)
Supplement: S2 Table — (DOCX) [file pone.0280485.s006.docx]

**S2 Table.** Data distribution of Five-fold cross-validation for deep learning.

| Patient | 1 | 2 | | 3 | 4 | | 5 | | 6 | 7 | 8 | 9 |
| --- | --- | --- | --- | --- | --- | --- | --- | --- | --- | --- | --- | --- |
| Number of data | 13 | 6 | | 26 | 17 | | 13 | | 10 | 8 | 14 | 13 |
| Fold#1 | Train | | | | | | | | | Validation | | Test |
| Fold#2 | Train | | | | | | | | Validation | | Test | Train |
| Fold#3 | Train | | | | | | | Validation | | Test | Train | |
| Fold#4 | Train | | | | | Validation | | | Test | Train | | |
| Fold#5 | Train | | Validation | | | Test | | Train | | | | |
