# Supplementary material for: Left ventricle segmentation in transesophageal echocardiography images using a deep neural network
Source: PLoS One. 2023 Jan 20;18(1):e0280485. doi: 10.1371/journal.pone.0280485 (PMC9858054; doi:10.1371/journal.pone.0280485)
Supplement: S3 Table — (DOCX) [file pone.0280485.s007.docx]

**S3 Table.** Formula of the metrics.

| Dice coefficient | $\frac{2TP}{2TP+FP+FN}$ |
| --- | --- |
| IoU | $\frac{TP}{TP+FP+FN}$ |
| Recall | $\frac{TP}{TP+FN}$ |
| Precision | $\frac{TP}{TP+FP}$ |
|  |  |
